# The role of preexisting analgesic use and self-efficacy for continued use of analgesics among patients with persistent low back pain

**DOI:** 10.1186/s12998-025-00612-0

**Published:** 2025-10-28

**Authors:** M. P. Tabul, A. Kongsted, J. Hartvigsen, M. S. Johansson

**Affiliations:** 1 Private practice of chiropractic, Køge, Denmark; 2 Center for Muscle and Joint Health, Department of Sports Science and Clinical Biomechanics, University of Southern Denmark, Campusvej 55, Odense M, 5250 Odense, Denmark; 3 Chiropractic Knowledge Hub, Odense, Denmark; 4 Research Unit of General Practice, Department of Public Health, University of Southern Denmark, Odense, Denmark

**Keywords:** Self-efficacy, Analgesics, Self-management, Low back pain, Coping

## Abstract

**Background:**

Various analgesics are frequently prescribed by physicians and used by patients with low back pain (LBP) despite limited effect on pain and disability and risk of side effects. Current knowledge on how psychological measures and self-management interventions influence analgesic use is limited. We investigated if analgesic use changed after participating in a patient education and exercise therapy program (GLA:D® Back), to what extent analgesic use and self-efficacy at baseline were potential determinants of analgesic use at the end of the program, and, to what extent improvement in self-efficacy from before to after the intervention modified the relationship between analgesic use at baseline and follow-up.

**Methods:**

We used data from the Danish GLA:D^®^ Back registry collected from March 28, 2018, until October 16, 2023. Potential determinants were self-reported baseline analgesic use and self-efficacy (the Arthritis Self Efficacy Scale pain subscale). The outcome was analgesic use at 3 months follow-up. We used logistic regression to investigate associations and effect modification.

**Results:**

Among 4721 included participants, 34% of those using analgesics at baseline (n = 942) discontinued this at 3 months follow-up. Analgesic use at baseline was associated with increased odds of analgesic use at follow-up (odds ratio [OR]: 9.79, 95% confidence interval [CI]: 7.88, 12.15), and higher levels of self-efficacy at baseline was associated with decreased odds of analgesic use at follow-up (OR: 0.85, 95% CI: 0.81, 0.89). Improved self-efficacy, obtained during the program, reduced the risk of analgesic use at follow-up from 15 to 6% and from 76 to 54% among participants with and without baseline analgesic use respectively.

**Conclusions:**

Patients using analgesics when initiating care were more likely to use analgesics three months later, while those having high levels of self-efficacy were less likely. Improved self-efficacy during the program reduced the absolute risk of analgesic use following the intervention to a larger extent among those using analgesics at baseline compared to those without baseline use. Further investigation is needed to confirm whether these findings reflect causal effects.

**Supplementary Information:**

The online version contains supplementary material available at 10.1186/s12998-025-00612-0.

## Introduction

Supported self-management plays a key role in patient-centred care in most chronic health conditions [1]. It refers to the individual's ability to manage symptoms, treatments, physical and psychological consequences, and lifestyle changes inherent in living with a chronic condition [2]. Engagement in self-management strategies is influenced by self-efficacy [3], defined as “the belief in one’s capability to organize and execute the courses of action required to produce given attainments” [4–6]. Low back pain (LBP) is a global public health challenge being the leading cause for years lived with disability worldwide [7–9]. Current clinical guidelines for non-specific LBP recommend an individualized, multimodal treatment approach that enhances the patient’s self-management [10–12]. Specifically, emphasizing care that improves self-efficacy and function, and reduces catastrophizing and fear avoidance [11, 12].

Recent evidence-based clinical guidelines only recommend certain analgesics with consideration. For example, the WHO guideline for management of chronic primary LBP conditionally recommends non-steroidal anti-inflammatory drugs (NSAIDs) for people under 60 years and recommend against the use of opioids, gabapentinoids, serotonin-norepinephrine reuptake inhibitors (SNRIs), tricyclic antidepressants (TCAs), and muscle relaxants [12]. Nonetheless, analgesics are commonly prescribed by physicians and widely used by individuals with LBP [13]. This despite limited evidence of an effect on pain and disability [14, 15], and documented side effects including liver damage from paracetamol [16–18], increased risk of gastrointestinal, renal, and cardiovascular events from non-steroidal anti-inflammatory drugs [19–21], and risk of addiction, overdose, and death from opioids [7, 22]. Although the relationship may not be causal, analgesic use for musculoskeletal pain is associated with higher pain intensity [23], worsened disability and negative illness perception [7], and a poor prognosis [24]. Analgesic use can be considered a passive coping strategy by relying on external agents to control pain [25], which has been linked to higher pain levels, disability, and depression across chronic pain conditions [7, 26]. This underlines the importance of providing patients with alternatives to pain medication.

Increasing patients’ self-management through better self-efficacy may lead to more active coping strategies [3, 27, 28] and potentially reduced analgesic use. However, knowledge on if and how self-efficacy influences analgesic use is scarce. Thus, the overall aim of this study was to investigate the role of self-efficacy in continued use of analgesics from before to after participation in a patient education and exercise therapy program (GLA:D^®^ Back) focussed on improving self-management strategies among patients with persistent LBP. Specifically, we aimed to investigate 1) whether patients change their analgesic use from before to after the intervention, 2) if self-efficacy at baseline affects analgesic use at follow up, and 3) whether improvement in self-efficacy from before to after the intervention modifies the effect of baseline analgesic use on future analgesic use.

## Methods

### Data sources and study population

This cohort study is a secondary analysis based on prospectively collected data from the GLA:D^®^ Back patient registry collected from March 28, 2018, until October 16, 2023. GLA:D^®^ Back is a group-based standardized patient education and exercise therapy program for patients with persistent or recurrent LBP. Patients were invited to participate if a clinician assessed that the patient would benefit from improved self-management skills. The program consists of an individual baseline assessment and goal setting followed by two 1-h sessions of group-based patient education and 8 weeks of supervised exercises (1-h sessions twice per week). The patient education is based on a behavioural approach, and addresses causes and the clinical course of back pain, common symptoms, purpose of imaging, treatment options, pain explanations, and management of pain. The exercise sessions include a warm-up, clinician-guided exercises focused on reestablishing natural movement and promoting confidence in back movements, and optional stretch exercises. Each exercise contains four levels of advancement. During the exercise session, the clinician encourages participants to explore variations in movements to find ways that work for the individual. The clinician also delivers core messages from the patient education to support helpful pain beliefs and self-efficacy while exercising [28].

Data were collected across private chiropractic and physical therapy clinics and municipality-based rehabilitation programs in Denmark. Out-of-pocket expenses for patients varied depending on whether the individual held private health insurance and whether the clinic was private or part of a municipal rehabilitation service [28].

The clinicians, consisting of mainly physiotherapists and chiropractors, involved in this study were certified GLA:D^®^ Back practitioners who had completed a 2-day training course aimed at standardizing both the treatment procedures and data collection. Study participants were asked to fill out an electronic questionnaire at baseline, 3-, 6-, and 12-months follow-up via REDcap, provided and supported by the Odense Patient data Explorative Network (OPEN) [29]. Only data from baseline and the 3-months follow-up were used in this study.

### Variables

#### Potential determinants: analgesic use and self-efficacy at baseline

Analgesic use at baseline was assessed by asking “Are you currently taking medication for your lower back pain?” with the possible answers “No”, “Yes, prescription medicine” or “Yes, over the counter medicine”. In May 2020 the option “Yes, both prescription and over the counter medicine” was added. We recoded the responses to “Yes” (indicating any use) or “No”. In Denmark, over-the-counter analgesics includes paracetamol and NSAIDs, while commonly prescribed analgesics include opioids, gabapentinoids, SNRIs, TCAs, and muscle relaxants, in addition to paracetamol and NSAIDs. These are hence the drugs we consider as analgesics in this context.

Self-efficacy was assessed using the Arthritis Self Efficacy Scale (ASES) Questionnaire. ASES is a questionnaire developed for patients with osteoarthritis or rheumatoid arthritis. ASES consists of questions that assess patients’ self-efficacy across three areas: managing pain, physical function, and control of other symptoms. Each item is scored from 1 to 10 where higher scores indicate greater levels of self-efficacy and a sum score is calculated for each subscale, also ranging from 1 to 10 [30]. In this study, we used the pain subscale and did not include the “control of other symptoms”-subscale due to collinearity between the two subscales. The questions for the pain subscale were:

(1) How certain are you that you can decrease your pain quite a bit? (2) How certain are you that you can continue most of your daily activities? (3) How certain are you that you can keep your pain from interfering with your sleep? (4) How certain are you that you can that you can make a small-to-moderate reduction in your back pain by using methods other than taking extra medication? (5) How certain are you that you can make a large reduction in your low back pain by using methods other than taking extra medication?

#### Effect modifier: change in self-efficacy

The change in self-efficacy was calculated as the difference between the ASES score at 3-months follow-up and the ASES score at baseline (i.e., a positive change score reflects improved self-efficacy). ASES has good internal consistency and test–retest reliability, but sensitivity is unknown [30]. Further, studies found sufficient reliability and validity in comparison to relevant health outcomes in a mixed group of chronic patients [31] and in patients with fibromyalgia [32]. Due to a non-linear relationship between change in self-efficacy and analgesic use at 3 months follow-up, we categorized the self-efficacy change score into “no positive change” (change score ≤ 0) and “positive change” (change score > 0).

#### Outcome: analgesic use

Analgesic use at the 3-month follow-up was assessed using the same question and responses as at baseline.

#### Potential confounders

Potential confounders included age, sex, level of education, back pain intensity, back-related disability, presence of comorbidities, and quality of life regarding general health.

- Level of education was assessed by asking “What is your highest formal education?” with the possible responses: “No completed formal education”, “Elementary school”, “High school”, “Vocational education”, “Short higher education”, “Intermediate higher education”, “Long higher education”, and “Other education”. We grouped the education levels into the following three categories: i) No vocational education consisting of “no completed formal education”, “elementary school” and “other education”; ii) Vocational education; and iii) Higher education consisting of “short”, “intermediate“, and “long higher education”.
- Back pain intensity was assessed with the question: “When you have had back pain during the past week, what were the typical pain intensity on a scale from 0 to 10, where 0 is “No pain at all”, and 10 is “Worst pain imaginable”?”[33].
- Back-related disability was measured with the Danish translation of the Oswestry Disability Index, which is a self-administered questionnaire that assesses 10 items reflecting the participant’s current function [34]. Each item is rated from 0 to 5 and the scores are calculated as a proportional sum score (0–100), with a high score reflecting a high level of disability [34]. If 5 or more items were not answered, the sum score was considered missing.
- Comorbidities were assessed by questions regarding the presence of 14 diseases (diabetes, osteoporosis, cardiovascular disease, hypertension, rheumatoid arthritis, osteoarthritis, metabolic disorder, asthma, migraine, chronic inflammatory bowel disease, cancer, chronic obstructive pulmonary disease, neurological disease [e.g., sclerosis or Parkinson’s], and other). Initially, it was reported by the clinicians, and after a revision of the questionnaire on May 15, 2020, it was reported by the patients. We used a variable reflecting the presence of one or more comorbidities (yes vs. no).
- Quality of life regarding general health was assessed by the following question from the Short Form 36 questionnaire: “In general, would you say your health is?” with responses on a 1–5 scale, where 1 indicated “Excellent” and 5 indicated “Poor” [28, 35].

### Statistical analysis

Participant characteristics were described using medians with first and third quartiles (Q1, Q3) and frequencies with percentages (overall, stratified by analgesic use at baseline and missing data) based on dataset with incomplete data (i.e., before multiple imputation). The ‘missing’ column (Table 1) includes observations with no data on analgesic use at baseline.

**Table 1:** Characteristics of 4721 participants in the final study population separated by analgesic use at baseline

Characteristic	All N = 4721 n (%) / median (Q1, Q3)	No analgesic use at baseline n = 1963 n (%) / median (Q1, Q3)	Analgesic use at baseline n = 2636 n (%) / median (Q1, Q3)	Missing data on analgesic use at baseline n = 122 n (%) / median (Q1, Q3)
*Sex*				
Male	1591 (34)	789 (40)	766 (29)	36 (30)
Female	3130 (66)	1174 (60)	1870 (71)	86 (70)
Missing	0	0	0	
Age (years)	59.00 (50.00, 67.00)	57.00 (48.00, 67.00)	60.00 (51.00, 68.00)	59.00 (46.50, 66.00)
Missing	0	0	0	0
*Analgesic use at 3 months follow-up*				
Yes	1303 (41)	156 (11)	1138 (65)	9 (29)
No	1909 (59)	1264 (89)	623 (35)	22 (71)
Missing	1509	543	875	91
Presence of comorbidities	3481 (82)	1341 (76)	2092 (86)	48 (81)
Missing	465	190	212	63
Back pain intensity (NRS)	6.00 (4.00, 7.00)	5.00 (3.00, 6.00)	6.00 (5.00, 8.00)	5.00 (2.75, 7.00)
Missing	63	6	7	50
Baseline self-efficacy score (ASES)	6.94 (5.40, 8.20)	7.60 (6.20, 8.74)	6.40 (5.00, 7.66)	6.76 (6.27, 7.22)
Missing	311	89	116	106
Baseline self-rated Quality of Life (SF-36)	3.00 (3.00, 4.00)	3.00 (2.00, 3.00)	3.00 (3.00, 4.00)	3.00 (2.00, 3.00)
Missing	294	78	114	102
Baseline disability level (ODI)	24.00 (16.00, 33.33)	17.78 (10.00, 24.44)	28.89 (20.00, 37.78)	24.00 (16.00, 28.00)
Missing	235	52	82	101
*Level of education*				
No vocational education	875 (19)	329 (17)	525 (20)	21 (22)
Vocational education	1216 (26)	483 (25)	710 (27)	23 (24)
Higher education	2016 (55)	1134 (58)	1119 (52)	52 (54)
Missing	93	17	50	26
3 months follow-up self-efficacy score (ASES)	7.12 (5.32, 8.40)	7.66 (6.04, 8.92)	6.40 (4.96, 8.02)	6.60 (5.00, 7.91)
Missing	1640	598	946	96
*Change in self-efficacy from baseline to 3 months follow-up*				
Positive change	1434 (48)	644 (48)	782 (47)	8 (73)
No positive change	1581 (52)	695 (52)	883 (53)	3 (27)
Missing	1706	624	971	111
Back pain intensity (NRS)	3.00 (2.00, 6.00)	3.00 (2.00, 5.00)	4.00 (3.00, 6.00)	3.00 (2.00, 5.00)
Missing	1432	518	825	89

N/n, number of observations; Q1 and Q3, first and third quartile; NRS, numerical rating scale; ASES, Arthritis Self-efficacy Scale; SF-36, Short Form 36 questionnaire; ODI; Oswestry Disability Index. Proportions do not include the number of missing values

We performed multivariate imputation by chained equations for missing data in self-efficacy, analgesic use, and in the potential confounders at baseline and follow-up using the ‘mice’ package [36] in R [37].

First, we determined the proportion of participants who changed their analgesic use from before to after the intervention. We then investigated if analgesic use at baseline was associated with analgesic use at 3-months follow-up using a logistic regression model (crude and adjusted for self-efficacy at baseline and all potential confounders defined above).

Secondly, we investigated whether baseline self-efficacy was associated with analgesic use at 3 months follow-up using a logistic regression model (crude and adjusted for analgesic use at baseline and all confounders).

Lastly, we investigated if the association between analgesic use at baseline and at 3-months follow-up was modified by changes in self-efficacy from baseline to 3-months follow-up by fitting a logistic regression model with analgesic use at baseline (yes vs. no), change in self-efficacy (categorized; “positive change” vs. “no positive change”), and their interaction term (crude and adjusted for baseline self-efficacy score and all confounders). Based on this model, we calculated i) odds ratios (OR) with 95% confidence intervals (CI) for the main effects and the interaction term (i.e., to investigate multiplicative interaction), and, to investigate the presence of an additive interaction, we calculated ii) the absolute risk (average predicted probability) of analgesic use at follow-up for each combination of analgesic use at baseline and change in self-efficacy, iii) risk differences (reference group: no analgesic use at baseline and no positive change in self-efficacy), and iv) a measure of interaction on the additive scale using the following formula:

$${p}_{11}-{p}_{10}-{p}_{01}+{p}_{00},$$

where ${p}_{11}$ denotes the risk of analgesic use at follow-up given baseline analgesic use and a positive change, ${p}_{10}$ denotes the risk given baseline analgesic use and no positive change, ${p}_{01}$ denotes the risk given no baseline analgesic use and a positive change, and ${p}_{00}$ denotes no baseline analgesic use and no positive change [38].

For all models, we investigated independence of errors, linearity between continuous variables and the outcomes, multicollinearity, and strongly influential observations.

As a sensitivity analysis, we compared the results based on imputed data with those based on a complete case analysis.

All statistical analyses were conducted using RStudio [37] running R version 4.3.1 [37].

## Results

**Fig. 1:**
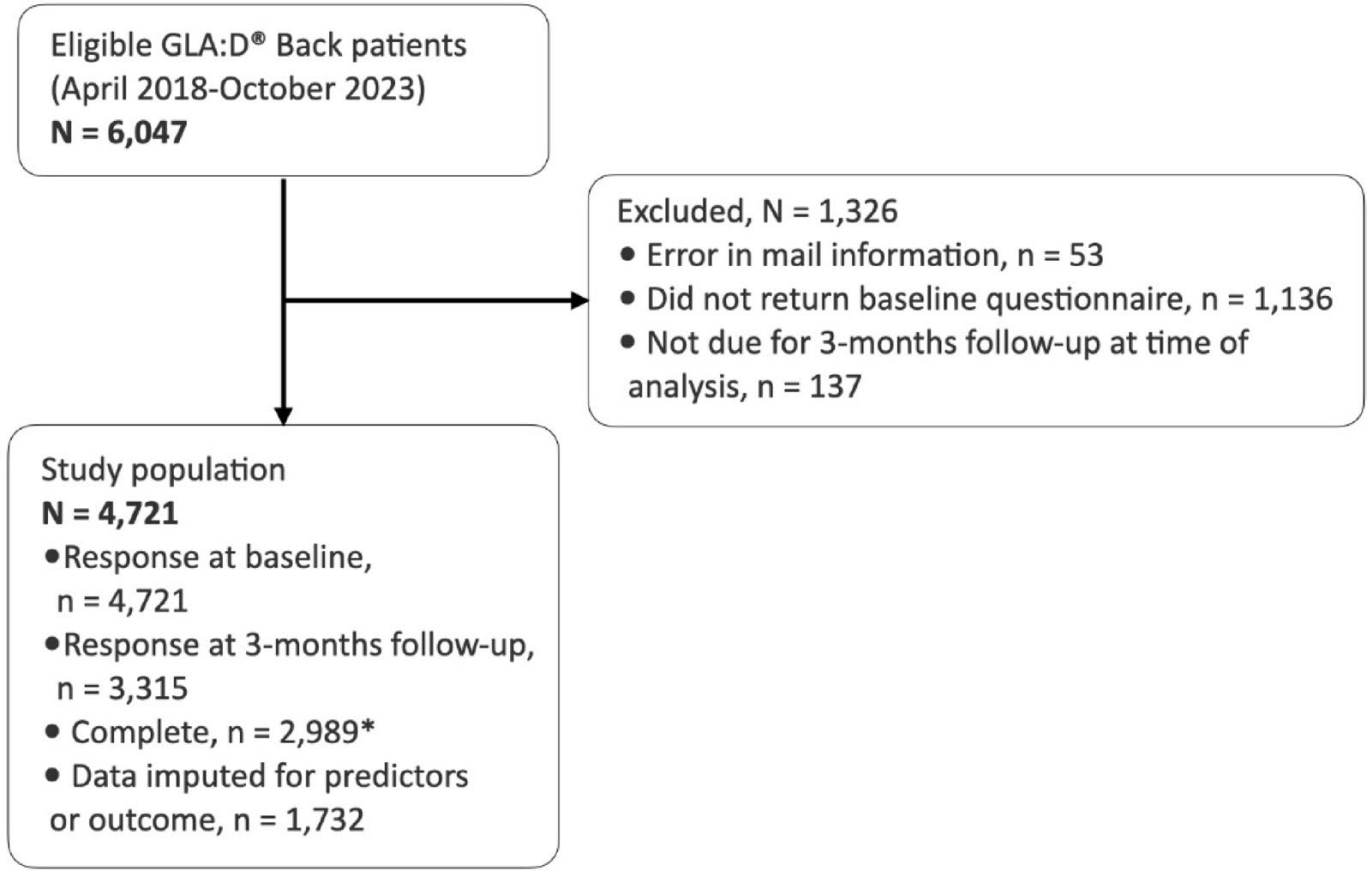
Formation of study population. N/n, number of observations. * Complete cases defined as having completed baseline and 3 months follow-up information regarding analgesic use and baseline self-efficacy

Out of 6047 eligible GLA:D^®^ Back participants, 4721 gave consent to participate and returned the baseline questionnaire (Fig. 1). Of these, 70% (3315/4721) filled out the questionnaire at 3 months follow-up (Fig. 1). The study population consisted of 66% women with a median age of 59 years (Q1: 50, Q3: 67) (Table 1). At baseline, 56% (2636/4721) of the population used analgesics (Table 1). Patients with analgesic use at baseline had higher scores in pain and disability, lower self-efficacy, and a larger proportion of them were females and had one or more comorbidities at baseline compared to those not using analgesics at baseline. Overall, those not reporting analgesic use at baseline had similar characteristics to the rest of the sample (Table 1).

Participants with missing values in the predictors and outcome were slightly younger, a larger proportion had a lower educational level, and slightly more reported baseline analgesic use compared to those with complete data (Supplementary File 1, Table S1).

Among those using analgesics at baseline, 66% continued to use and 34% discontinued their analgesic use at 3 months follow-up (Table 2). Among non-users at baseline, 89% stayed non-users while 11% started to use analgesics at the 3 months follow-up. In the regression analyses, participants who used analgesics at baseline were more likely to use analgesics at 3-months follow-up compared to non-users at baseline (adjusted OR: 9.79, 95% CI: 7.88, 12.15; Table 3). Higher self-efficacy at baseline was associated with reduced odds for analgesic use at 3 months follow-up (adjusted OR: 0.85, 95% CI: 0.81, 0.89; Table 3).

**Table 2:** Changes in analgesic use from baseline to 3 months follow-up

	Analgesic use at 3 months follow-up n (%)	No analgesic use at 3 months follow-up n (%)	Total N (%)
Analgesic use at baseline	1795 (66%)	908 (34%)	2703 (100%)
No analgesic use at baseline	212 (11%)	1806 (89%)	2018 (100%)
Total	2007 (43%)	2714 (57%)	4721 (100%)

**Table 3:** Crude and adjusted association between analgesic use and self-efficacy at baseline and analgesic use at 3 months follow-up

Predictor	Crude OR (95% CI)	Adjusted OR (95% CI)
Analgesic use at baseline (yes vs. no)	15.45 (12.35, 19.34)	9.79 (7.88, 12.15)
ASES pain score at baseline (1–10)	0.71 (0.68, 0.74)	0.85 (0.81, 0.89)

OR, odds ratio; CI, confidence interval; ASES, Arthritis Self-Efficacy Scale

The adjusted model included both analgesic use at baseline and self-efficacy at baseline, and age, sex, level of education, back pain intensity, back-related disability, presence of comorbidities, and quality of life regarding general health

**Fig. 2:**
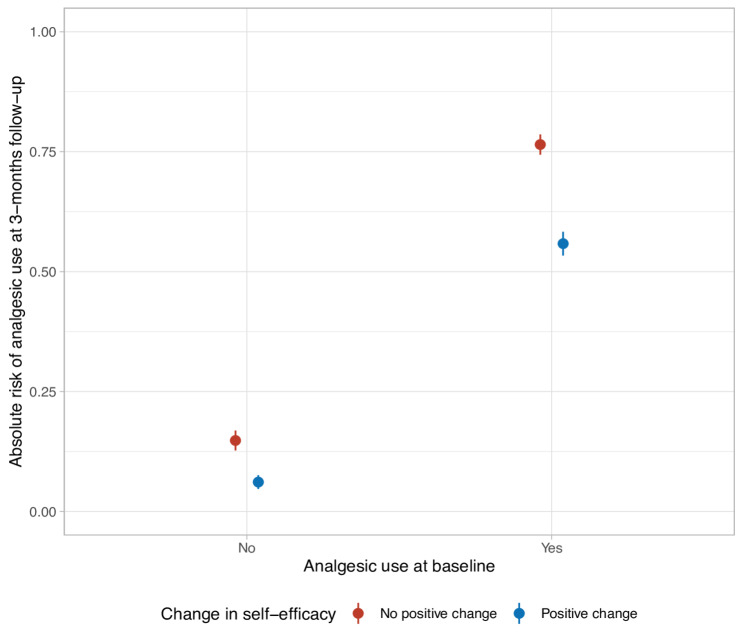
Absolute risk (average predicted probability) of analgesic use at 3 months follow-up for each combination of analgesic use at baseline and change in self-efficacy

On the multiplicative (odds ratio) scale, we found no evidence of effect modification of change in self-efficacy (crude model, *p* = 0.95; adjusted model, *p* = 0.60; Table 4). Among patients with no baseline analgesic use, the absolute risk of analgesic use at follow-up was 15% among those with no positive change and 6% among those with a positive change in self-efficacy. In comparison, among patients using analgesics at baseline, the corresponding absolute risks were 76% and 54% (Fig. 2). Using “no baseline analgesic use” and “no positive change in self-efficacy” as reference, the risk difference was 61% for baseline analgesic use alone (i.e., 0.76–0.15 = 0.61), -9% for a positive change in self-efficacy alone, and 39% for both baseline analgesic use and a positive change in self-efficacy (Table 5). This reflects a negative additive interaction of -13% (95% CI: -0.18, -0.08) on the risk difference scale. That is, a positive change in self-efficacy attenuated the risk of analgesic use at follow-up more among those with an analgesic use at baseline than among those without baseline use.

**Table 4:** Investigation of whether change in self-efficacy from baseline to 3-months follow-up modified the association between analgesic use at baseline and analgesic use at follow-up

Predictor	Crude OR (95% CI)	Adjusted OR (95% CI)
Analgesic use at baseline (yes vs. no)	17.47 (13.46, 22.68)	10.77 (8.38, 13.86)
Change in self-efficacy (positive change vs. no positive change)	0.37 (0.24, 0.57)	0.22 (0.14, 0.35)
Interaction-term: Analgesic use at baseline (yes) x Change in self-efficacy (positive change)	1.01 (0.66, 1.56)	1.12 (0.71, 1.77)

OR, odds ratio; CI, confidence interval; ASES, Arthritis Self-Efficacy Scale

Crude and adjusted association between analgesic use and self-efficacy at baseline and at 3 months follow-up, and between self-efficacy at baseline and analgesic use at 3 months follow-up. The adjusted model included self-efficacy at baseline, age, sex, level of education, back pain intensity, back-related disability, presence of comorbidities, and quality of life regarding general health

**Table 5:** Risk differences of analgesic use at 3 months follow-up across baseline analgesic use and change in self-efficacy status

Change in self-efficacy	No baseline analgesic use RD (95% CI)	Baseline analgesic use RD (95% CI)
No positive change	0.00 (Ref.)	0.61 (0.57, 0.64)
Positive change	-0.09 (-0.13, -0.05)	0.39 (0.34, 0.45)

RD, risk difference; CI, confidence interval; Ref., reference group

Risk differences estimated using logistic regression model including analgesic use at baseline, self-efficacy at baseline, change in self-efficacy from baseline to 3-months follow-up, age, sex, level of education, back pain intensity, back-related disability, presence of comorbidities, and quality of life regarding general health

The complete case analyses showed similar results to the analyses on imputed data, except that the interaction term was statistically significant (*p* = 0.04; Supplementary file 2).

## Discussion

Our results provide insights about the role of preexisting analgesic use and self-efficacy for continued use of analgesics among patients with persistent LBP in primary care. Most participants did not change their analgesic use from before to after the standardized patient education and exercise therapy programme, however, 35% did discontinue analgesic use. People who used analgesics when entering the care program had substantially higher odds of using analgesics after the intervention, while those with higher self-efficacy at baseline had lower odds. Although we did not find a positive change in self-efficacy to modify the association between analgesic use at baseline and follow-up on the multiplicative (log-odds) scale, we found it to attenuate the absolute risk of continued analgesic use among analgesic users at baseline (i.e., negative additive interaction).

We found a strong relationship between baseline analgesic use and use at follow-up, which is consistent with previous studies investigating predictors of prolonged analgesic use after joint replacement surgery. Although in a different context, they report similar associations of baseline analgesic use being a strong predictor of future analgesic use [39–42]. This suggests that it might be a universal challenge to stop analgesic use once it is initiated. Interestingly, our study found that most participants maintained consistent analgesic use despite a pain reduction at a group level over time (Table 1), indicating that analgesic use might be part of a self-management strategy or maybe becomes habitual. The relatively short follow-up period precludes us from making conclusions about long-term effects.

Our findings suggest that self-efficacy levels at baseline influences future analgesic use, and that improved self-efficacy can reduce the (absolute) risk of future analgesic use among patients using analgesics at baseline (i.e., continued use). Thus, achieving higher levels of self-efficacy may be of importance for discontinued analgesic use. In a study investigating patterns of analgesic use, self-efficacy, and pain intensity among rheumatological patients, no association between regularity of analgesic use and self-efficacy was found when adjusting for pain severity [43]. The authors did, however, find that patients with higher perceived self-efficacy reported less pain. However, recommendations for pain management in inflammatory arthritis suggest taking analgesics [44], whereas for LBP, guidelines discourage it as a long-term strategy. Therefore, the patterns of analgesic use might differ between the groups.

### Methodological considerations

Strengths of our study include a high response rate (70%) in a large population receiving standardized care for persistent LBP, validated measurements, and the use of multivariate imputation by chained equations for missing data (with similar results when compared to a complete case analysis; Tables S2). Patients who agree to participate in a patient education and exercise therapy program may have higher levels of self-efficacy compared to patients not choosing to participate in such interventions [3, 45]. Higher self-efficacy levels among arthritis patients have been found to be associated with a higher likelihood to adhere to an exercise program [46–48], which might also be the case among LBP patients. Future studies could avoid this potential bias by collecting data from, for example, general practitioners unrelated to offering specific interventions for larger variation in self-efficacy levels, and thus a more general understanding of the association between self-efficacy and analgesic use. That would, however, result in a less homogeneous study population.

The change in self-efficacy score was dichotomised due to a non-linear relationship with analgesic use at 3-months follow-up. We acknowledge that this generally results in loss of information, but we consider the observed change scores to have been presented in a clinically relevant way in the categorical variable. Polynomials were not used, although it has been suggested as a solution, since we considered this to have hampered interpretation and communication to clinicians.

Our data on analgesic use did not allow us to differentiate between over the counter and prescription analgesics. This is a limitation since some prescription drugs will be more difficult to discontinue (e.g., opioids) than over the counter drugs (e.g., paracetamol).

When collecting the data, the ASES pain subscale could have been replaced with the comprehensive ASES-8 questionnaire covering all self-efficacy domains. However, the data collection in the GLA:D Back registry is conducted for several purposes, limiting what can be asked within each domain addressed. Future studies could include more detailed information about confounders such as type of comorbidities (severity, medical treatment, psychological/psychiatric or somatic comorbidities).

As described in the Methods section, the responses to the question about analgesic use were modified in 2020. We chose to use data on analgesic use from before and after this modification since only relying on the data after 2020 would have resulted in a small study sample. However, this was done at the cost of more detailed data (i.e., being able to differentiate between over the counter and prescribed analgesics). We acknowledge that our measure of analgesic use is rough and that some participants may use analgesics sporadically while others may use it daily. Future studies should preferably use more detailed data on types of analgesics, their dose, and frequency of use, for example by using prescription registry data. Nonetheless, we believe that the current data can be used as a starting point to investigate potential causal associations between analgesic use and self-efficacy at baseline and continued analgesic use at follow-up, which can inform more complex causal models.

Finally, we adjusted for several important confounders but acknowledge the risk of residual confounding. It should hence be recognized that the observed effects may not be causal. Also, given the study design, the decrease in analgesic users after the intervention (34% discontinuing use) should not be interpreted as a causal effect of the intervention.

### Clinical implications

Our study findings help health care providers target their communication and treatment away from initiating analgesic use and highlights the importance of supporting people to discontinue use when initiated potentially preventing prolonged analgesic use and associated side effects. It also points to high self-efficacy for pain and symptom control being potentially important for reducing analgesic use. This aligns well with previous findings that improved self-efficacy may mediate treatment effects in LBP [49].

## Conclusions

Patients using analgesics before starting a standardized patient education and exercise therapy program for LBP were more likely to use analgesics three months later, while those having high levels of self-efficacy were less likely. Improved self-efficacy during the program reduced the absolute risk of analgesic use following the intervention to a larger extent among those using analgesics at baseline compared to those without baseline use. Our findings indicate that improved self-efficacy may, potentially, be useful to prevent prolonged analgesic use. However, further investigation is needed to confirm whether these findings reflect causal effects.

## Supplementary Information

Below is the link to the electronic supplementary material.

Supplementary Material 1

Supplementary Material 2

## Data Availability

The dataset supporting the conclusions of this article is available from the Chiropractic Knowledge Hub (contact Orla Lund Nielsen, e-mail: o.nielsen@kiroviden.sdu.dk). The coding used for the analysis is available upon request to the corresponding author. The GLA:D^®^ registry has been approved by the Danish Data Protection Agency (SDU; 10.084). According to the Danish Data Protection Act patient consent was not required for this analysis as personal data was processed exclusively for research and statistical purposes.

## Abbreviations

LBP: Low back pain
OPEN: Odense patient data explorative network
ASES: Arthritis self efficacy scale
WHO: World health organization
NSAIDs: Non-steroidal anti-inflammatory drugs
SNRIs: Serotonin-norepinephrine reuptake inhibitors
TCAs: Tricyclic antidepressants
Q1, Q3: First and third quartiles
OR: Odds ratio
CI: Confidence interval
n: Number of observations
RD: Risk difference
