# Supplementary material for: The role of preexisting analgesic use and self-efficacy for continued use of analgesics among patients with persistent low back pain
Source: Chiropr Man Therap. 2025 Oct 28;33:51. doi: 10.1186/s12998-025-00612-0 (PMC12570813; doi:10.1186/s12998-025-00612-0)
Supplement: Supplementary file 1 — Supplementary Material 1 [file 12998_2025_612_MOESM1_ESM.pdf]

Supplementary file 1

Table S1. Characteristics of participants stratified by missing values on predictors and outcome

**Characteristic**	**Participants without missing values on predictors and outcome N = 2,989 n (%) / median (Q1, Q3)**	**Participants with missing values on predictors and outcome n = 1,732 n (%) / median (Q1, Q3)**
Sex		
Male	1,026 (34)	565 (33)
Female	1,963 (66)	1,167 (67)
Missing	0	0
Age (years)	60.00 (51.00, 67.00)	58.00 (47.00, 67.00)
Missing	0	0
Analgesic use at baseline		
Yes	1,655 (55)	981 (61)
No	1,334 (45)	629 (39)
Missing	0	122
Analgesic use at 3 months follow-up		
Yes	1,211 (41)	92 (41)
No	1778 (59)	131 (59)
Missing	0	1,509
Presence of comorbidities	2,284 (82%)	1,197 (82%)
Missing	196	269
Back pain intensity (NRS)	6.00 (4.00, 7.00)	6.00 (4.00, 7.00)
Missing	5	58
Baseline self-efficacy score (ASES)	7.00 (5.50, 8.20)	6.60 (5.14, 8.00)
Missing	0	311
3 months follow-up self-efficacy score (ASES)	7.12 (5.32, 8.40)	6.78 (5.18, 8.00)
Missing	0	1,640
Change in self-efficacy from baseline to 3 months follow-up		
Positive change	1,417 (47)	17 (65)
No positive change	1,572 (53)	9 (35)
Missing	0	1,706
Baseline self-rated Quality of Life (SF-36)	3.00 (2.00, 4.00)	3.00 (3.00, 4.00)
Missing	19	275
Baseline disability level (ODI)	22.22 (14.00, 32.00)	26.00 (17.78, 34.82)
Missing	1	234
Level of education		
No vocational education	512 (19)	363 (25)

Vocational education	754 (29)	462 (31)
Higher education	1,361 (52)	655 (44)
Missing	362	252
N/n, number of observations; Q1 and Q3, first and third quartile; NRS, numerical rating scale; ASES, Arthritis Self-efficacy Scale; SF-36, Short Form 36 questionnaire; ODI; Oswestry Disability Index. Proportions do not include number of missing values.		
