# Supplementary material for: The role of preexisting analgesic use and self-efficacy for continued use of analgesics among patients with persistent low back pain
Source: Chiropr Man Therap. 2025 Oct 28;33:51. doi: 10.1186/s12998-025-00612-0 (PMC12570813; doi:10.1186/s12998-025-00612-0)
Supplement: Supplementary file 2 — Supplementary Material 2 [file 12998_2025_612_MOESM2_ESM.pdf]

Supplementary file 2

Complete case analysis

**Table S2**. Changes in analgesic use from baseline to 3 months follow-up			
	**Analgesic use at 3 months follow-up**	**No analgesic use at 3 months follow-up**	**Total**
Analgesic use at baseline	985 (64%)	545 (36%)	1,530 (100%)
No analgesic use at baseline	137 (11%)	1,072 (89%)	1,209 (100%)
Total	1,122 (41%)	1,617 (59%)	2,739 (100%)

**Table S3**. Crude and adjusted association between analgesic use at baseline and analgesic use at 3 months follow-up, and between self-efficacy at baseline and analgesic use at 3 months follow-up		
	**Crude OR (95% CI)**	**Adjusted OR (95% CI)**
Analgesic use at baseline (yes/no)	14.14 (11.54, 17.44)	8.93 (7.18, 11.18)
ASES pain score at baseline (1-10)	0.71 (0.68, 0.75)	0.84 (0.80, 0.89)
OR, odds ratio; CI, confidence interval; ASES, Arthritis Self-Efficacy Scale. N = 2,739		

**Table S4**. Investigation of whether change in self-efficacy from baseline to 3-months follow-up modified the association between analgesic use at baseline and analgesic use at follow-up		
**Predictor**	**Crude OR (95% CI)**	**Adjusted OR (95% CI)**
Analgesic use at baseline (yes vs. no)	14.14 (10.94, 18.43)	8.55 (6.47, 11.38)
Change in self-efficacy (positive change vs. no positive change)	0.30 (0.20, 0.45)	0.17 (0.11, 0.26)
Interaction-term: Analgesic use at baseline (yes) x Change in self-efficacy (positive change)	1.39 (0.88, 2.23)	1.68 (1.04, 2.77)
OR, odds ratio; CI, confidence interval; ASES, Arthritis Self-Efficacy Scale.<br>Crude and adjusted association between analgesic use and self-efficacy at baseline and at 3 months follow-up, and between self-efficacy at baseline and analgesic use at 3 months follow-up. The adjusted model included self-efficacy at baseline, age, sex, level of education, back pain intensity, back-related disability, presence of comorbidities, and quality of life regarding general health. N = 2,739		

Table S5. Risk differences of analgesic use at 3 months follow-up across baseline analgesic use and change in self-efficacy status

Change in self-efficacy	No baseline analgesic use RD (95% CI)	Baseline analgesic use RD (95% CI)
**No positive change**	0.00 (Ref.)	0.57 (0.53, 0.61)
**Positive change**	-0.11 (-0.14, -0.08)	0.37 (0.33, 0.42)

RD, risk difference; CI, confidence interval; Ref., reference group.
Risk differences estimated using logistic regression model including analgesic use at baseline, self-efficacy at baseline, change in self-efficacy from baseline to 3-months follow-up, age, sex, level of education, back pain intensity, back-related disability, presence of comorbidities, and quality of life regarding general health.
